# Antimicrobial Use in Hospitalised Patients with COVID-19: An International Multicentre Point-Prevalence Study

**DOI:** 10.3390/antibiotics11020176

**Published:** 2022-01-28

**Authors:** Lea Papst, Roberto Luzzati, Biljana Carević, Carlo Tascini, Nina Gorišek Miksić, Vera Vlahović Palčevski, Zorana M. Djordjevic, Omar Simonetti, Emanuela Sozio, Milica Lukić, Goran Stevanović, Davor Petek, Bojana Beović

**Affiliations:** 1 Department of Infectious Diseases, University Medical Centre Ljubljana, Zaloška cesta 2, 1000 Ljubljana, Slovenia; milica.lukic@kclj.si (M.L.); bojana.beovic@kclj.si (B.B.); 2 Faculty of Medicine, University of Ljubljana, Vrazov trg 2, 1000 Ljubljana, Slovenia; 3 Department of Infectious Diseases, Azienda Sanitaria Universitaria Giuliano Isontina, Via Giacomo Puccini 50, 34148 Trieste, Italy; roberto.luzzati@asugi.sanita.fvg.it (R.L.); omar.simonetti@asugi.sanita.fvg.it (O.S.); 4 Department of Hospital Epidemiology, University Clinical Centre of Serbia, Pasterova 2, 11000 Belgrade, Serbia; biljana.carevic@gmail.com (B.C.); goran_drste@yahoo.com (G.S.); 5 Infectious Diseases Clinic, Azienda Sanitaria Universitaria Friuli Centrale, Via Pozzuolo 33, 33100 Udine, Italy; carlo.tascini@asufc.sanita.fvg.it (C.T.); emanuela.sozio@gmail.com (E.S.); 6 Department of Infectious Diseases, University Medical Centre Maribor, Ljubljanska ulica 5, 2000 Maribor, Slovenia; nina.gorisekmiksic@ukc-mb.si (N.G.M.); davor.petek@ukc-mb.si (D.P.); 7 Unit of Clinical Pharmacology, Clinical Hospital Center Rijeka, Krešimirova ulica 42, 51000 Rijeka, Croatia; vera.vlahovicpalcevski@gmail.com; 8 Department of Hospital Infections Control, University Clinical Centre Kragujevac, Zmaj Jovina 30, 34000 Kragujevac, Serbia; drzorana.25@gmail.com

**Keywords:** COVID-19, antimicrobial use, multicentre, point-prevalence study

## Abstract

Studies suggest that the incidence of coinfections in patients with the coronavirus disease 2019 (COVID-19) is low, but a large number of patients receive antimicrobials during hospitalisation. This may fuel a rise in antimicrobial resistance (AMR). We conducted a multicentre point-prevalence survey in seven tertiary university hospitals (in medical wards and intensive care units) in Croatia, Italy, Serbia and Slovenia. Of 988 COVID-19 patients, 521 were receiving antibiotics and/or antifungals (52.7%; range across hospitals: 32.9–85.6%) on the day of the study. Differences between hospitals were statistically significant (χ^2^ (6, *N* = 988) = 192.57, *p* < 0.001). The majority of patients received antibiotics and/or antifungals within 48 h of admission (323/521, 62%; range across hospitals: 17.4–100%), their most common use was empirical (79.4% of prescriptions), and pneumonia was the main indication for starting the treatment (three-quarters of prescriptions). The majority of antibiotics prescribed (69.9%) belonged to the “Watch” group of the World Health Organization AWaRe classification. The pattern of antimicrobial use differed across hospitals. The data show that early empiric use of broad-spectrum antibiotics is common in COVID-19 patients, and that the pattern of antimicrobial use varies across hospitals. Judicious use of antimicrobials is warranted to prevent an increase in AMR.

## 1. Introduction

Antimicrobial resistance (AMR) is an inevitable consequence of antimicrobial use, making antimicrobial stewardship (AMS) an irreplaceable tool in the fight against increasing resistance [1]. Amidst the ever-increasing threat of AMR, the coronavirus disease 2019 (COVID-19) pandemic has shifted the focus of healthcare providers to the care of patients infected with severe acute respiratory syndrome coronavirus 2 (SARS-CoV-2). Since the pandemic began in 2020, nearly 270 million cases of COVID-19 have been confirmed, claiming more than 5.3 million lives worldwide [2].

Studies of bacterial and fungal infections in patients with COVID-19 suggest that the incidence of these infections is low, but a large number of patients receives antibiotics upon admission to hospital or during hospitalisation. Early bacterial coinfection has been reported in only 1.2–3.5% of patients with COVID-19 [3,4,5,6]. In rapid systematic reviews, bacterial or fungal infections have been identified in 6.9–8% of patients [7,8], with early bacterial coinfection being rare (3.5% of patients), and secondary bacterial/fungal infection occurring in 14.3% of patients [8]. Studies and systematic reviews reported the use of antimicrobials in up to 38.3–74.6% of patients [4,5,7,9,10].

It is still unclear how the pandemic and antibiotic use in patients with COVID-19 will affect AMR, as evidence-based data on AMR is still lacking [11]. Some studies have reported an increase in infections with multi-drug resistant microorganisms (MDR) [12,13,14,15], while others have found no increase in infections with MDR bacteria and fungi [6,16]. The differences suggest heterogeneous antibiotic use and infection control measures in COVID-19 patients in hospitals.

To better guide antimicrobial prescribing and adjust AMS programmes, more information is needed on antimicrobial use in patients with COVID-19 from different parts of the world. We conducted an international multicentre point-prevalence survey to collect comprehensive data on the characteristics and differences of antibiotic and antifungal prescribing in hospitalised patients with COVID-19 in various countries and hospitals in South-Eastern Europe.

## 2. Results

Of 988 COVID-19 patients, 521 were receiving antibiotics and/or antifungals on the day of the study (52.7%; range across hospitals: 32.9–85.6%). Differences between hospitals were statistically significant (χ^2^ (6, *N* = 988) = 192.57, *p* < 0.001). Use of antibiotics and antifungals was common in intensive care units (ICUs) (135/186, 72.6%; range across hospitals: 54.1–100%) as well as in medical wards (386/802, 48.1%; range across hospitals: 14.3–93.6%). The characteristics of patients who received antibiotic or antifungal therapy are shown in Table 1. The majority of patients were male (61.2%), with an even higher proportion of male patients in ICUs (73.3%). The median age was 69 years, and the most common chronic disease was arterial hypertension. Approximately 75% of patients on antibiotics and/or antifungals were receiving concomitant corticosteroids and supplemental oxygen. Approximately two-thirds of patients were receiving one antibiotic/antifungal, others two or more. The median levels of C-reactive protein (CRP) and procalcitonin (PCT) at the start of antimicrobial therapy were 86.7 mg/L and 0.2 μg/L, respectively. For chronic diseases as well as for CRP and WBC (white blood count), statistically significant differences between hospitals were observed (Table 1). In ICU, 71.9% of patients were intubated and mechanically ventilated (range across hospitals: 20–95%), 14.1% were pronated (range across hospitals: 0–50%), 40% were receiving vasoactive support (range across hospitals: 13.3–70%) and 0.74% were on extracorporeal membrane oxygenation (ECMO) (range across hospitals: 0–3.4%).

The majority of patients received an antimicrobial agent within 48 h of admission (323/521, 62%; range across hospitals: 17.4–100%), 70.2% in medical wards (271/386; range across hospitals: 23.5–100%) and 38.5% in ICUs (52/135; range across hospitals: 0–100%). A total of 743 antibiotics and antifungals was prescribed. Their use was most commonly empirical (79.4% of prescriptions) (Table 2). Targeted treatment was more common in ICUs, especially with antibiotics and antifungals started later in the course of hospitalisation (≥48 h after admission); 47% in ICUs vs. 31.3% in medical wards. Antibiotics and/or antifungals were prescribed for pneumonia in three-quarters of cases, followed by urinary tract infections (5.5% of prescriptions) and infections of no known origin (5.3% of prescriptions) (Table 3). While pneumonia was almost the only indication for antibiotics and/or antifungals being prescribed at the beginning of hospitalisation (88% of prescriptions), less than half of them were prescribed due to pneumonia in medical wards later in the hospitalisation (45.2%), with other hospital-acquired infections gaining importance. In ICUs pneumonia remained the main indication for treatment with antibiotics and/or antifungals (75.5% of prescriptions). According to the treating physicians, the reasons for starting antimicrobial therapy were clinical presentation in 85.4% of cases, laboratory findings in 72.2% and imaging in 62.6% of cases.

**Figure 1:**
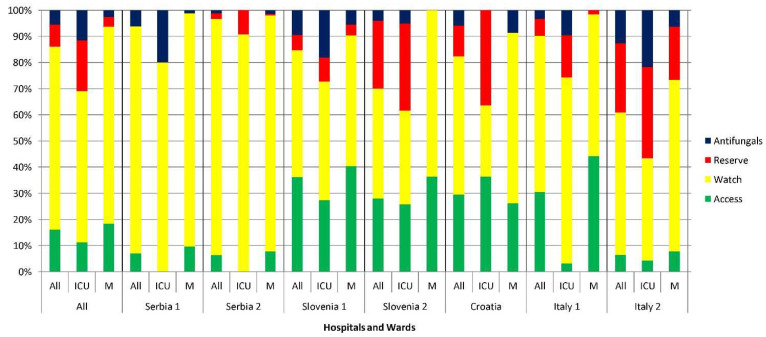
Use of antibiotics (according to AWaRe classification) and antifungals in various hospitals. All: all wards; ICU: intensive care unit; M: medical ward; Access: access antibiotics-penicillins, beta-lactams with beta-lactamase inhibitors, tetracyclines, trimethoprim with sulfametoxazole, aminoglycosides and metronidazole; Watch: watch antibiotics-cephalosporins (2nd–4th generation), antipseudomonal beta-lactams with beta-lactamase inhibitors, carbapenems, fluoroquinolones, macrolides and vancomycin; Reserve: reserve antibiotics-5th generation cephalosporins, polymyxins, glycylcyclins, oxazolidinones and lipopeptides.

The majority of antibiotics prescribed (69.9%) belonged to the “Watch” group of the World Health Organization (WHO) AWaRe classification: cephalosporins (2nd–4th generation), antipseudomonal beta-lactams with beta-lactamase inhibitors, carbapenems, fluoroquinolones, macrolides and vancomycin (Figure 1). In ICUs, antifungals and antibiotics from the “Reserve” group of the AWaRe classification (5th generation cephalosporins, polymyxins, glycylcyclins, oxazolidinones, lipopeptides, etc.) were prescribed more frequently than in medical wards; antifungals accounted for 11.6% and antibiotics from the “Reserve” group for 19.3% of antimicrobial prescriptions in ICUs. The pattern of antimicrobial use varied across hospitals. In some hospitals, antibiotics from the “Access” group (penicillins, beta-lactams with beta-lactamase inhibitors, tetracyclines, trimethoprim with sulfametoxazole, aminoglycosides, metronidazole, etc.) accounted for approximately one-third of prescriptions (including in ICUs), whereas in other hospitals their use was rare.

Analyses of microbiological samples and isolated microorganisms were performed only in patients receiving targeted therapy (Table 3). Targeted treatments with antibiotics and antifungals were based on the results of 114 positive samples. In the medical wards, 40.3% of the positive samples were urine cultures and 26.3% were blood cultures. In the ICUs, bronchoalveolar lavage (BAL) and tracheal aspirate each accounted for 38.6% of the positive samples. The most frequently isolated microorganisms were Gram-negative bacteria: enterobacterales and non-fermenting bacilli. *Escherichia coli* accounted for 22.8%, *Klebsiella* spp. for 15.8% and *Clostridioides difficile* for 10.5% of all isolates in the medical wards. In ICUs, the most common isolates were *Acinetobacter* spp., *Pseudomonas* spp., *Klebsiella* spp., MRSA and *Aspergillus* spp.

## 3. Discussion

We conducted an international multicentre point-prevalence study to gain information on antimicrobial prescribing in patients with COVID-19 to better guide antimicrobial stewardship in COVID-19 wards. Overall, 52.7% of patients were receiving antibiotics and/or antifungals on the day of the study. The therapy was empiric in most cases, i.e., prescribed on or shortly after admission, with broad-spectrum antibiotics usually used.

The percentage of patients receiving antibiotics and/or antifungals varied between hospitals (32.9–85.6%) and was higher in ICUs than in medical wards (72.6% vs. 48.1%). Reviews examining antimicrobial use in COVID-19 patients reported higher overall antimicrobial use (74.6% and 72% of patients) [7,10]. Prescription appears to vary between different hospitals and countries. In a study conducted in Scottish hospitals, 38.3% of COVID-19 patients were identified as receiving antibiotic therapy [9]; in a study from China 58% were so identified [17]; in some studies from the beginning of the pandemic, the percentage of patients receiving antibiotics was as high as 99% [18,19]. It is worth noting that the period of interest in our study was not the first wave, when uncertainty and common practice were in favour of antibiotic prescribing.

The majority of patients in our study received antimicrobial therapy early in the course of hospitalisation (62% in the first 48 h), but there were significant differences between hospitals: the range between hospitals was 17.4–100%. These results are consistent with other published studies. A study that examined coinfections and early antibiotic therapy in hospitalised COVID-19 patients at four Dutch hospitals found that 60.1% of patients received antibiotics within the first 24 h of admission. Similar to our study, antimicrobial use varied between hospitals: 33.3–72.2% [5]. Another multi-hospital study conducted in the United States of America found early empiric antibiotic use in 56.6% of hospitalised COVID-19 patients, again with wide variation between hospitals (27–84%) [4].

The vast majority of prescriptions was empirical (79.4%), especially early in the hospitalisation. Targeted treatment was more common in ICUs, probably due to the sampling of the lower respiratory tract. The most common indication for therapy with antibiotics and/or antifungals was pneumonia (three-quarters of all prescriptions), especially at the beginning of hospitalisation. Later, it remained the most important indication for antimicrobials, but, especially on medical wards, other hospital-acquired infections, such as urinary tract infections, skin and soft tissue infections and intraabdominal infections, also became an important factor.

The high percentage of antimicrobials stands in stark contrast to the low incidence of coinfections and secondary infections in COVID-19 patients reported in the literature; the overall rate of bacterial or fungal infections is approximately 7–8% [6,7]. Early bacterial coinfections in particular appear to be low, only appearing in approximately 3% of COVID-19 patients, according to studies [4,6,8]. Secondary infections that develop during hospitalisation also appear to be rare, occurring in 4.7–14.3% of patients [6,8].

Broad-spectrum antibiotic therapy was generally used in both medical wards and ICUs. This is consistent with the published literature, in which treatment with cephalosporins, fluoroquinolones, macrolides, beta-lactams with beta-lactamase inhibitors and carbapenems is most commonly described [7,10,18,19]. “Reserve” antibiotics against MDR microorganisms were commonly prescribed in ICUs (19.3% of prescriptions), with resistant bacteria such as *Pseudomonas* spp. and *Acinetobacter* spp. (mainly carbapenem-resistant) accounting for 42.1% of positive ICU samples. MDR bacteria, particularly Gram negative bacilli, are an important cause of hospital-acquired infections in critically ill COVID-19 patients, most commonly of ventilator-associated pneumonia [20,21]. Several outbreaks of infections with MDR bacteria were described during the pandemic waves [14,15,22].

Antifungal drugs were used most frequently in ICUs (11.6% of prescriptions), which was expected due to the fungal infections that occur after the use of high doses of corticosteroids and other immunosuppressive therapies used to treat critically ill COVID-19 patients [23]. In our study, *Aspergillus* spp. was isolated in 14% of positive ICU samples, with 74.1% of ICU patients receiving corticosteroids on the day of the study. One of the first descriptions of an increased rate of pulmonary aspergillosis in COVID-19 patients (CAPA) was performed in Italy, with approximately 30% of patients admitted to ICU diagnosed with probable CAPA, which was associated with high mortality [24]. A study conducted across five ICUs in France showed a lower incidence of secondary fungal infections. A total of 4.8% of patients had a probable/putative invasive pulmonary mould infection, and clinically irrelevant colonisation or false-positive fungal tests were observed in 17.2% of patients [25].

Enterobacterales were most commonly isolated in medical wards (42.9% of positive samples), with urine and blood cultures accounting for 66.6% of positive samples. *C. difficile* was also an important pathogen (10.5% of positive samples), probably due to treatment with broad-spectrum antibiotics. Other studies reported *Streptococcus pneumoniae*, *S. aureus*, *E. coli*, *Haemophilus influenzae* and *Pseudomonas* spp. as common pathogens, which were isolated mainly from respiratory, blood or urine cultures [4,6,8]. In our study, positive respiratory samples were a rarity in medical wards, even though pneumonia was the main indication for antimicrobial therapy. Due to the fact that we did not record negative samples, we cannot distinguish between the low use of respiratory cultures and the low yield of respiratory samples. Studies show that sputum cultures are performed relatively infrequently (7.7–11.4% of patients) due to aerosolisation concerns and the fact that the majority of patients with COVID-19 have a dry, non-productive cough [4,5].

Patterns of antimicrobial use varied among hospitals. Statistically significant differences in patient characteristics show that antimicrobials were prescribed to diverse groups of patients, which may account for differences in prescription. However, various approaches to antimicrobial therapy are more likely to reflect differences in local antimicrobial susceptibilities and antimicrobial treatment guidelines for patients with COVID-19, which are usually in line with local guidelines for pneumonia [26]. There are differences in antimicrobial prescription among the four different countries included in the study. The total consumption of antibacterial agents in DDDs per 1000 inhabitants per day in 2019 was 18.8 in Croatia, 21.7 in Italy, 13.0 in Slovenia and 28.65 in Serbia, with “Watch” category antibiotics accounting for 30% of all antibiotics prescribed in Slovenia and approximately 40% in the other three countries [27,28]. In some hospitals in our study, broad-spectrum antibiotic therapy was initiated on admission, while in others a more prudent approach to antimicrobial therapy was adopted. In accordance with local antimicrobial susceptibilities, hospitals in Serbia and Italy more frequently prescribed antibiotics from the “Watch” and “Reserve” lists of the AWaRe classification. As a consequence of differences in antimicrobial prescribing practices, the impact of COVID-19 on AMR is likely to vary from hospital to hospital, with an increase in AMR expected in hospitals where empirical broad-spectrum antibiotic therapy was frequently prescribed.

The data from our study and the published literature suggest that the use of most broad-spectrum antibiotics is probably unwarranted and unnecessary, especially early in hospitalisation for COVID-19, when the incidence of coinfection is remarkably low. Antimicrobial stewardship teams implementing local guidelines should encourage prescribers to use a more restrained approach. Antibiotics should not be routinely prescribed to patients on admission without evidence of bacterial coinfection. However, it may be difficult to distinguish between severe COVID-19 and bacterial coinfection, especially in critically ill patients. In these patients, microbiological sampling should be encouraged to either guide or discontinue antimicrobial treatment, based on its results. The role of biomarkers such as procalcitonin (PCT) may also be of value. Levels of PCT below 0.1 μg/L have been shown to have a negative predictive value of 98.3% [4]. Other studies have shown that antibiotics can be safely withheld or discontinued in COVID-19 patients with PCT levels below 0.25 μg/L in the absence of other features of bacterial infections [29,30]. The median value of PCT in our study (0.2 μg/L) suggests that many prescribed antimicrobials were probably unnecessary. Higher values of PCT may be more difficult to interpret, because they may have occurred as a result of coinfection or severe COVID-19 [31]. Typical chest radiologic findings in patients with COVID-19 may also help to distinguish between pneumonia due to COVID-19 and bacterial pneumonia [32,33]. Thus, the results of imaging studies are another tool that can help physicians decide whether to treat with antibiotics. As a result of our study, AMS activities were encouraged in the participating hospitals.

Our study provides a comprehensive overview of antimicrobial prescription practices in seven university hospitals caring for COVID-19 patients. It is the first point-prevalence study to examine prescription in medical wards and ICUs in a large cohort of hospitalised COVID-19 patients in several countries and the first report of antimicrobial use during COVID-19 pandemic from the region. Due to its point-prevalence design, it can only provide a snapshot of practices in a particular time window during the pandemic. As information about COVID-19 has grown rapidly, treatment strategies have also evolved over time. Point-prevalence studies can also be influenced by fluctuating prescriptional trends, e.g., biphasic patterns recorded in earlier waves [34]. Due to the fact that information about patient characteristics was collected only for patients receiving antimicrobials, analysis of differences in patient populations was possible only for patients on antimicrobials and not for all hospitalised patients with COVID-19. We analysed only positive samples sent for microbiological diagnostics; therefore, assessment of the frequency of sampling and proportion of positivity was not possible. We also did not specifically investigate the appropriateness of the antimicrobials prescribed, which is another limitation of our study.

## 4. Materials and Methods

Our multicentre point-prevalence study took place from 11 February 2021 to 15 April 2021 in seven tertiary university hospitals in four countries (Croatia, Italy, Serbia, Slovenia). It was conducted in medical wards and ICUs where patients with confirmed COVID-19 were cared for.

Each COVID-19 ward was audited once and on a single day. All patients 18 years of age and older with confirmed COVID-19 who were hospitalised on the ward at eight o’clock in the morning and were, at the time, receiving systemic antimicrobial therapy were included in the study. The study was conducted by hospital-based physicians. Data were collected about the following drugs: antibiotics for systemic use (Anatomical Therapeutic Chemical (ATC) classification J01), antifungals for systemic use (ATC J02 and D01BA), drugs for the treatment of tuberculosis (ATC J04A), antibiotics for the treatment of intestinal infections (ATC A07AA) and antiparasitic drugs that can be used as antibacterial agents (ATC P01AB).

Two forms were used for data collection (Supplementary Materials, Supplements S1 and S2). The ward form was used to collect information about the number of beds, the number of patients and the number of ventilated patients on the ward (denominator data). Patient forms were used for inpatients receiving antimicrobial therapy (nominator data). For each patient, patient characteristics, days of hospitalisation, days of antimicrobial therapy, indications for antibiotic or antifungal use, type of antimicrobial and its dosage, biomarker levels and radiological findings, use of supplemental oxygen and treatment of COVID-19 were recorded. Antibiotic and antifungal treatment was classified as either prophylactic (medical or surgical) or therapeutic (empirical or targeted). In cases of targeted treatment, microbiological data were also collected. All samples collected for microbiological diagnostics were taken at the discretion of the treating physicians. For ICU patients, information about mechanical ventilation, vasoactive support, pronation and ECMO was also included.

Descriptive statistics were performed using Microsoft Excel. Separate analyses were performed for medical wards and intensive care units and for antimicrobials prescribed within 48 h and after 48 h of hospitalisation. The 2019 WHO AWaRe Classification was used for the analysis of prescribed antibiotics [35]. For analysing differences between hospitals, χ^2^ or Fisher’s exact test were used for categorical variables and ANOVA for numeric variables. A Bonferroni correction for multiple comparisons was used. Statistical analysis was performed using SPSS Statistics for Windows, version 28.0.

## 5. Conclusions

In summary, the data from our study show that early empiric use of broad-spectrum antibiotics is common in COVID-19 patients, and that the pattern of antimicrobial use varies from hospital to hospital. The main indication for initiation of antimicrobial therapy is pneumonia. The widespread use of last-line antibiotics identified in some settings, combined with the heavy burden of hospitalised COVID-19 patients, may lead to a substantial increase in AMR. Judicious use of antimicrobials is warranted to prevent an increase in AMR, given the low rates of coinfection and secondary infection reported in the literature. AMS teams should adapt local guidelines accordingly, monitor their implementation and assist treating physicians when real-life dilemmas arise.

## Figures and Tables

**Table 1:** Characteristics of patients receiving antibiotics and/or antifungals.

	Total (*N* = 521) % (Range across Hospitals, %)	Differences between Hospitals		Medical Wards (*N* = 386) % (Range across Hospitals, %)	ICUs (*N* = 135) % (Range across Hospitals, %)
		Χ^2^/Fisher’s Exact *t*-Test (6, *N* = 521)	ANOVA		
Sex		0.67, *p* = 0.995			
Male	61.2% (58.6–65.2%)			57% (44.4–59.1%)	73.3% (59.1–100%)
Female	38.8% (34.8–41.4%)			43% (40.9–55.6%)	26.7% (0–40.9%)
Age in years, median (IQR), (range across hospitals)	69 (17) (62–75)		F (6, 514) = 7.21, *p* < 0.001	69 (19) (61–79)	68 (12.5) (62.5–75.5)
Days of hospitalisation, median (IQR), (range across hospitals)	8 (10) (5–16)		F (6, 514) = 15.89, *p* < 0.001	7 (9) (5–19)	12 (11) (7–14.5)
Comorbidities					
Hypertension	64.3% (40.7–88.5%)	71.82, *p* < 0.001		61.9% (36.4–90.9%)	71.1% (44.4–94.4%)
Other cardiovascular diseases	36.3% (13.6–62.3%)	63.45, *p* < 0.001		36.8% (12.1–70.5%)	34.8% (14.8–70.5%)
Diabetes	29.9% (17.3–54.8%)	34.41, *p* < 0.001		30.6% (16.7–59.1%)	28.2% (0–59.1%)
Chronic obstructive lung disease	15.2% (6.9–32.9%)	40.92, *p* < 0.001		13.2% (0–34.1%)	20.7% (10–44.4%)
Other lung diseases	9% (0–13.6%)	15.03, *p* = 0.014		8.3% (0–18%)	11.1% (0–22.2%)
Neurological disease	7.7% (0–15.1%)	23.14, *p* < 0.001		8% (0–19.7%)	6.7% (0–16.7%)
Mental disorder	7.1% (0–24.6%)	50, *p* < 0.001		6.7% (0–25.6%)	18.2% (0–22.2%)
Liver disease	3.7% (0–17.2%)	34.2, *p* < 0.001		2.6% (0–22.2%)	6.7% (0–15%)
Chronic kidney disease	8.6% (0–22.2%)	41.58, *p* < 0.001		8.8% (1.4–22.2%)	8.2% (0–25%)
Immunocompromised	9.4% (3.3–21%)	16.18, *p* = 0.009		9.3% (4.7–22.2%)	9.6% (0–30%)
Treatment of COVID-19					
Antiviral agents	12.5% (0–11.1%)	71.33, *p* < 0.001		13% (0–11.1%)	11.1% (0–25%)
Corticosteroids	74.3% (34.8–87.9%)	64.25, *p* < 0.001		74.4% (33.3–89%)	74.1% (16.7–100%)
Supplemental oxygen	75.2% (53.1–91.8%)	62.44, *p* < 0.001		69.7% (42.6–88.4%)	91.1% (66.7–100%)
Number of antibiotics/antifungals					
1	65.6% (51.7–77.8%)			71.2% (58.1–85.2%)	49.6% (33.3–63.6%)
2	26.3% (14.8–32.8%)			25.4% (11.5–41.9%)	28.9% (11.1–50%)
≥3	8.1% (3.5–24.1%)			3.4% (0–11.8%)	21.5% (9.1–35%)
Laboratory findings					
CRP in mg/L, median (IQR), (range across hospitals)	86.7 (107.8) (56–149)		F (6, 512) = 7.72, *p* < 0.001	75.8 (97.1) (51.1–118)	120.5 (133.7) (53.2–153)
PCT in μg/L, median (IQR), (range across hospitals)	0.2 (0.4) (0.2–0.4)			0.2 (0.3) (0.1–0.3)	0.2 (0.7) (0.1–0.6)
Leukocyte count in 10^9^/L, median (IQR), range across hospitals	7.5 (6) (5.8–11.4)		F (6, 509) = 13.42, *p* < 0.001	7 (5.3) (5.6–9.9)	9.9 (8.3) (6.6–10.6)

ICUs: intensive care units; IQR: interquartile range; COVID-19: coronavirus disease 19; CRP: C-reactive protein; PCT: procalcitonin.

**Table 2:** Antibiotic and antifungal use by type of treatment and indication.

	Total (*N* = 743)	Medical Wards (*N* = 510)	ICUs (*N* = 233)	≤48 h			>48 h		
				Total (*N* = 425)	Medical Wards (*N* = 344)	ICUs (*N* = 81)	Total (*N* = 317)	Medical Wards (*N* = 166)	ICUs (*N* = 151)
Type of treatment									
Prophylactic use; medical	13 (1.8%)	8 (1.6%)	5 (2.2%)	8 (1.9%)	5 (1.5%)	3 (3.7%)	5 (1.6%)	3 (1.8%)	2 (1.3%)
Therapeutic use; empirical	590 (79.4%)	439 (86.1%)	151 (64.8%)	400 (94.1%)	328 (95.4%)	72 (87.7%)	189 (59.6%)	111 (66.9%)	78 (51.7%)
Therapeutic use; targeted	140 (18.8%)	63 (12.4%)	77 (33.1%)	17 (4%)	11 (3.2%)	6 (7.4%)	123 (38.8%)	52 (31.3%)	71 (47%)
Indication									
Pneumonia	564 (75.9%)	379 (74.3%)	185 (79.4%)	374 (88%)	304 (88.4%)	70 (86.4%)	189 (59.6%)	75 (45.2%)	114 (75.5%)
Bloodstream infection	11 (1.5%)	7 (1.4%)	4 (1.7%)	2 (0.5%)	0	2 (2.5%)	9 (2.8%)	7 (4.2%)	2 (1.3%)
Central-line associated bloodstream infection	7 (0.9%)	4 (0.8%)	3 (1.3%)	0	0	0	7 (2.2%)	4 (2.4%)	3 (2%)
Urinary tract infection	41 (5.5%)	37 (7.3%)	4 (1.7%)	14 (3.3%)	14 (4.1%)	0	27 (8.5%)	23 (13.9%)	4 (2.7%)
Skin and soft tissue infection	15 (2%)	15 (2.9%)	0	5 (1%)	5 (1.5%)	0	10 (3.2%)	10 (6%)	0
Intra-abdominal infection	22 (3%)	20 (3.9%)	2 (0.9%)	5 (1%)	5 (1.5%)	0	17 (5.4%)	15 (9%)	2 (1.3%)
Bone and joint infection	7 (0.9%)	4 (0.8%)	3 (1.3%)	0	0	0	7 (2.2%)	4 (2.4%)	3 (2%)
Unknown site of infection	39 (5.3%)	16 (3.1%)	23 (9.9%)	12 (2.8%)	6 (1.7%)	6 (7.4%)	27 (8.5%)	10 (6%)	17 (11.3%)
Other	37 (5%)	28 (5.5%)	9 (3.9%)	13 (3.1%)	10 (2.9%)	3 (3.7%)	24 (7.6%)	18 (10.8%)	6 (4%)

ICUs: intensive care units; ≤48 h: antimicrobials started within 48 h of admission; >48 h: antimicrobials started more than 48 h after admission.

**Table 3:** Positive microbiology samples and isolated microorganisms.

	Total (*N* = 114)	Medical Wards (*N* = 57)	ICUs (*N* = 57)
Positive Microbiology Samples			
Blood culture	19 (16.7%)	15 (26.3%)	4 (7%)
Sputum	2 (1.8%)	1 (1.8%)	1 (1.8%)
Tracheal aspirate	24 (21.1%)	2 (3.5%)	22 (38.6%)
BAL, mini BAL	23 (20.2%)	1 (1.8%)	22 (38.6%)
Urine culture	28 (17.5%)	23 (40.3%)	5 (8.8%)
Other	18 (15.8%)	15 (26.3%)	3 (5.3%)
Isolated microorganisms			
MSSA	13 (11.4%)	3 (5.3%)	10 (17.5%)
MRSA	3 (2.6%)	1 (1.8%)	2 (3.5%)
CoNS	2 (1.8%)	2 (3.5%)	0
*Streptococcus* spp.	5 (4.4%)	1 (1.8%)	4 (7%)
*Enterococcus faecalis*	7 (6.1%)	6 (10.5%)	1 (1.8%)
*Enterococcus faecium*	5 (4.4%)	2 (3.5%)	3 (5.3%)
*Escherichia coli*	20 (17.5%)	13 (22.8%)	7 (12.3%)
ESBL-producing *E. coli* CR *E. coli*	4 (3.5%)	1 (1.8%)	3 (5.3%)
	1 (0.9%)	1 (1.8%)	0
*Klebsiella* spp.	19 (16.7%)	9 (15.8%)	10 (17.5%)
ESBL–producing *Klebsiella* spp. CR *Klebsiella* spp.	5 (4.4%)	1 (1.8%)	4 (7%)
	5 (4.4%)	2 (3.5%)	3 (5.3%)
*Proteus mirabilis*	10 (8.7%)	6 (10.5%)	4 (7%)
*Pseudomonas* spp.	12 (10.5%)	5 (8.8%)	7 (12.3%)
CR *Pseudomonas* spp.	1 (0.9%)	0	1 (1.8%)
*Acinetobacter* spp.	19 (16.7%)	2 (3.5%)	17 (29.8%)
CR *Acinetobacter* spp.	10 (8.7%)	0	10 (17.5%)
Anaerobes	9 (7.9%)	8 (14%)	1 (1.8%)
*Clostridioides difficile* (toxin positive)	7 (6.1%)	6 (10.5%)	1 (1.8%)
*Aspergillus* spp.	9 (7.9%)	1 (1.8%)	8 (14%)
*Candida* spp.	6 (5.3%)	2 (3.5%)	4 (7%)
Other	14 (12.3%)	3 (5.3%)	11 (19.3%)

ICUs: intensive care units; BAL: bronchoalveolar lavage; MSSA: methicillin-susceptible *Staphylococcus aureus*; MRSA: methicillin-resistant *S. aureus*; CoNS: coagulase-negative staphylocci; ESBL: extended-spectrum beta-lactamase; CR: carbapenem-resistant.

## Data Availability

Available on request.

## Supplementary Materials

The following supporting information can be downloaded at: https://www.mdpi.com/article/10.3390/antibiotics11020176/s1, Supplement S1: Ward form; Supplement S2: Patient form.

## References

[1] World Health Organization Global Action Plan on Antimicrobial Resistance. https://www.who.int/publications-detail-redirect/9789241509763.

[2] World Health Organization WHO Coronavirus (COVID-19) Dashboard. https://covid19.who.int/.

[3] Hughes S., Troise O., Donaldson H., Mughal N., Moore L.S.P. (2020). Bacterial and fungal coinfection among hospitalized patients with COVID-19: A retrospective cohort study in a UK secondary-care setting. Clin. Microbiol. Infect..

[4] Vaughn V.M., Gandhi T.N., Petty L.A., Patel P.K., Prescott H.C., Malani A.N., Ratz D., McLaughlin E., Chopra V., Flanders S.A. (2021). Empiric antibacterial therapy in community-onset bacterial coinfection in patients hospitalized with Coronavirus disease 2019 (COVID-19): A multi-hospital cohort study. Clin. Infect. Dis..

[5] Karami Z., Knoop B.T., Dofferhoff A.S.M., Blaauw M.J.T., Janssen N.A., van Apeldoorn M., Kerckhoffs A.P.M., van de Maat J.S., Hoogerwerf J.J., ten Oever J. (2021). Few bacterial co-infections but frequent empiric antibiotic use in the early phase of hospitalized patients with COVID-19: Results from a multicentre retrospective cohort study in The Netherlands. Infect. Dis..

[6] Garcia-Vidal C., Sanjuan G., Moreno-García E., Puerta-Alcalde P., Garcia-Pouton N., Chumbita M., Fernandez-Pittol M., Pitart C., Inciarte A., Bodro M. (2021). Incidence of co-infections and superinfections in hospitalized patients with COVID-19: A retrospective cohort study. Clin. Microbiol. Infect..

[7] Rawson T.M., Moore L.S.P., Zhu N., Ranganathan N., Skolimowska K., Gilchrist M., Satta G., Cooke G., Holmes A. (2020). Bacterial and fungal coinfection in individuals with coronavirus: A rapid review to support COVID-19 antimicrobial prescribing. Clin. Infect. Dis..

[8] Langford B.J., So M., Raybardhan S., Leung V., Westwood D., MacFadden D.R., Soucy J.-P.R., Daneman N. (2020). Bacterial co-infection and secondary infection in patients with COVID-19: A living rapid review and meta-analysis. Clin. Microbiol. Infect..

[9] Seaton R.A., Gibbons C.L., Cooper L., Malcolm W., McKinney R., Dundas S., Griffith D., Jeffreys D., Hamilton K., Choo-Kang B. (2020). Survey of antibiotic and antifungal prescribing in patients with suspected and confirmed COVID-19 in Scottish hospitals. J. Infect..

[10] Langford B.J., So M., Raybardhan S., Leung V., Soucy J.-P.R., Westwood D., Daneman N., MacFadden D.R. (2021). Antibiotic prescribing in patients with COVID-19: Rapid review and meta-analysis. Clin. Microbiol. Infect..

[11] Monnet D.L., Harbarth S. (2020). Will coronavirus disease (COVID-19) have an impact on antimicrobial resistance?. Euro Surveill..

[12] Kampmeier S., Tönnies H., Correa-Martinez C.L., Mellmann A., Schwierzeck V. (2020). A nosocomial cluster of vancomycin resistant enterococci among COVID-19 patients in an intensive care unit. Antimicrob. Resist. Infect. Control.

[13] Posteraro B., Torelli R., Vella A., Leone P.M., De Angelis G., De Carolis E., Ventura G., Sanguinetti M., Fantoni M. (2020). Pan-echinocandin-resistant *Candida glabrata* bloodstream infection complicating COVID-19: A fatal case report. J. Fungi.

[14] Nori P., Szymczak W., Puius Y., Sharma A., Cowman K., Gialanella P., Fleishner Z., Corpuz M., Torres-Isasiga J., Bartash R. (2020). New Delhi metallo-beta-lactamase producing Enterobacterales infections in New York City COVID-19 patients. Int. J. Antimicrob. Agents.

[15] Tiri B., Sensi E., Marsiliani V., Cantarini M., Priante G., Vernelli C., Martella L.A., Costantini M., Mariottini A., Andreani P. (2020). Antimicrobial stewardship program, COVID-19, and infection control: Spread of carbapenem-resistant *Klebsiella pneumoniae* colonization in ICU COVID-19 patients. What did not work?. J. Clin. Med..

[16] Contou D., Claudinon A., Pajot O., Micaëlo M., Longuet Flandre P., Dubert M., Cally R., Logre E., Fraissé M., Mentec H. (2020). Bacterial and viral co-infections in patients with severe SARS-CoV-2 pneumonia admitted to a French ICU. Ann. Intensive Care.

[17] Guan W.-J., Ni Z.-Y., Hu Y., Liang W.-H., Ou C.-Q., He J.-X., Liu L., Shan H., Lei C.-L., Hui D.S.C. (2020). Clinical characteristics of Coronavirus disease 2019 in China. N. Eng. J. Med..

[18] Nori P., Cowman K., Chen V., Bartash R., Szymczak W., Madaline T., Punjabi Katiyar C., Jain R., Aldrich M., Weston G. (2021). Bacterial and fungal coinfections in COVID-19 patients hospitalized during the New York City pandemic surge. Infect. Control Hosp. Epidemiol..

[19] Cao J., Tu W.-J., Cheng W., Yu L., Liu Y.-K., Hu X., Liu Q. (2020). Clinical features and short-term outcomes of 102 patients with Coronavirus disease 2019 in Wuhan, China. Clin. Infect. Dis..

[20] Baiou A., Elbuzidi A.A., Bakdach D., Zaqout A., Alarbi K.M., Bintaher A.A., Ali M.M.B., Elarabi A.M., Ali G.A.M., Daghfal J. (2021). Clinical characteristics and risk factors for the isolation of multi-drug-resistant Gram-negative bacteria from critically ill patients with COVID-19. J. Hosp. Infect..

[21] Graselli G., Scaravilli V., Mangioni D., Scudeller L., Alagna L., Bartoletti M., Bellani G., Biagioni E., Bonfanti P., Bottino N. (2021). Hospital-acquired infections in critically ill patients with COVID-19. Chest J..

[22] Patel A., Emerick M., Cabunoc M.K., Williams M.H., Preas M.A., Schrank G., Rabinowitz R., Luethy P., Johnson J.K., Leekha S. (2021). Rapid spread and control of multidrug-resistant Gram-negative bacteria in COVID-19 patient care units. Emerg. Infect. Dis..

[23] Machado M., Valerio M., Álvarez-Uría A., Olmedo M., Veintimilla C., Padilla B., De la Villa S., Guinea J., Escribano P., Ruiz-Serrano M.J. (2021). Invasive pulmonary aspergillosis in the COVID-19 era: An expected new entity. Mycoses.

[24] Bartoletti M., Pascale R., Cricca M., Rinaldi M., Maccaro A., Bussini L., Fornaro G., Tonetti T., Pizzilli G., Francalanci E. (2021). Epidemiology of invasive pulmonary aspergillosis among COVID-19 intubated patients: A prospective study. Clin. Infect. Dis..

[25] Fekkar A., Lampros A., Mayaux J., Poignon C., Demeret S., Constantin J.-M., Marcelin A.-G., Monsel A., Luyt C.-E., Blaize M. (2021). Occurrence of invasive pulmonary fungal infections in patients with severe COVID-19 admitted to the ICU. Am. J. Respir. Crit. Care Med..

[26] Beović B., Doušak M., Ferreira-Coimbra J., Nadrah K., Rubulotta F., Belliato M., Berger-Estilita J., Ayoade F., Rello J., Erdem H. (2020). Antibiotic use in patients with COVID-19: A ‘snapshot’ Infectious Diseases International Research Initiative (ID-IRI) survey. J. Antimicrob. Chemother..

[27] European Centre for Disease Prevention and Control Antimicrobial Consumption Database (ESAC-Net). http://www.ecdc.europa.eu./en/antimicrobial-consumption/surveillance-and-disease-data/database.

[28] Tomas A., Pavlović N., Stilinović N., Horvat O., Paut-Kusturica M., Dugandžija T., Tomić Z., Sabo A. (2021). Increase and change in the pattern of antibiotic use in Serbia (2010–2019). Antibiotics.

[29] Williams E.J., Mair L., de Silva T.I., Green D.J., House P., Cawthron K., Gillies C., Wigfull J., Parsons H., Partridge D.G. (2021). Evaluation of procalcitonin as a contribution to antimicrobial stewardship in SARS-CoV-2 infection: A retrospective cohort study. J. Hosp. Infect..

[30] Peters C., Williams K., Un E.A., Little L., Saad A., Lendrum K., Thompson N., Weatherley N.D., Pegden A. (2021). Use of procalcitonin for antibiotic stewardship in patients with COVID-19: A quality improvement project in a district general hospital. Clin. Med..

[31] Zhou F., Yu T., Du R., Fan G., Liu Y., Liu Z., Xiang J., Wang Y., Song B., Gu X. (2020). Clinical course and risk factors for mortality of adult inpatients with COVID-19 in Wuhan, China: A retrospective cohort study. Lancet.

[32] Wong H.Y.F., Lam H.Y.S., Fong A.H.-T., Leung S.T., Chin T.W.-Y., Lo C.S.Y., Lui M.M.-S., Lee J.C.-Y., Chiu K.W.-H., Chung T.W.-H. (2020). Frequency and distribution of chest radiographic findings in patients positive for COVID-19. Radiology.

[33] Ye Z., Zhang Y., Wang Y., Huang Z., Song B. (2020). Chest CT manifestations of new coronavirus disease 2019 (COVID-19): A pictorial review. Eur. Radiol..

[34] Abelenda-Alonso G., Padullés A., Rombauts A., Gudiol C., Pujol M., Alvarez-Pouso C., Jodar R., Carratalà J. (2020). Antibiotic prescription during the COVID-19 pandemic: A biphasic pattern. Infect. Control Hosp. Epidemiol..

[35] World Health Organization 2019 WHO AWaRe Classification Database of Antibiotics for Evaluation and Monitoring of Use. https://www.who.int/publications-detail.redirect/WHOEMPIAU2019.11.

