# Supplementary material for: Antimicrobial Use in Hospitalised Patients with COVID-19: An International Multicentre Point-Prevalence Study
Source: Antibiotics (Basel). 2022 Jan 28;11(2):176. doi: 10.3390/antibiotics11020176 (PMC8868464; doi:10.3390/antibiotics11020176)
Supplement: Supplementary file 1 [file antibiotics-11-00176-s001.zip › Supplement S1.pdf]

Point-prevalence survey of antimicrobial and antifungal treatment in patients with covid-19

Ward form

date	
name of the ward*	
type of the ward	ward Intensive care unit
number of hospital beds at 8.00 a.m.	
number of patients at 8.00 a.m.	
number of intubated patients**	
number of ventilated patients**	

** name that is used in the hospital

**for intensive care units
