# Supplementary material for: Antimicrobial Use in Hospitalised Patients with COVID-19: An International Multicentre Point-Prevalence Study
Source: Antibiotics (Basel). 2022 Jan 28;11(2):176. doi: 10.3390/antibiotics11020176 (PMC8868464; doi:10.3390/antibiotics11020176)
Supplement: Supplementary file 1 [file antibiotics-11-00176-s001.zip › Supplement S2.pdf]

Point-prevalence survey of antimicrobial and antifungal treatment in patients with covid-19

Date	Patient's identificator	Ward	Days of hospitalization before PPS*:	age	sex	weight	height

**Chronic diseases**	**Indication for antibiotic/antifungal**	**1. antibiotic or antifungal drug**	**2. antibiotic or antifungal drug**	**3. antibiotic or antifungal drug**
arterial hypertension	pneumonia	Generic name	Generic name	Generic name
other cardiovascular diseases	urinary tract infection	days of hospitalisation before antibiotic/antifungal*	days of hospitalisation before antibiotic/antifungal*	days of hospitalisation before antibiotic/antifungal*
diabetes	bacteriemia	duration of treatment before PPS*	duration of treatment before PPS*	duration of treatment before PPS*
COPD	vascular line infection	application mode	application mode	application mode
other lung diseases	skin and skin structure infection	dose	dose	dose
neurological diseases<br>mental disorder	intra-abdominal infection	dose interval	dose interval	dose interval
liver diseases	unknown site of infection	empirical treatment	empirical treatment	empirical treatment
chronic kidney failure	bone	targeted treatment	targeted treatment	targeted treatment
immune deficiency	other	prophylaxis:<br>medical surgical	prophylaxis:<br>medical surgical	prophylaxis:<br>medical surgical

**treatment of COVID-19**			
remdesivir	other antivirals	corticosteroids (type), dose, duration to antibiotic	oxygen, fiO2

What triggered antibiotic/antifungal treatment: signs/symptoms laboratory tests imaging

**Laboratory tests at the start of antimicrobial**	C-rp	PCT	WBC

Chest x-ray at the start of antimicrobial (in patients with antibiotics for pneumonia): new pneumonia worsening

In the case of targeted treatment: isolated micro-organism

**type of specimen**	sputum	tracheal aspirate	BAL, mini-Bal	blood culture	urine culture	swab	biopsy	intraoperative swab	biomarkers for fungal infections	other

*count the days of introduction but not the day of PPS

**Intensive care unit**							
intubation	NIV	mechanical ventilation	FiO2	pronation	vasoactive support	haemodialysis	ECCMO
